# Use of quality‐of‐life instruments for people living with HIV: a global systematic review and meta‐analysis

**DOI:** 10.1002/jia2.25902

**Published:** 2022-04-09

**Authors:** Ying Zhang, Christine He, Tessa Peasgood, Emily S. G. Hulse, Christopher K. Fairley, Graham Brown, Richard Ofori‐Asenso, Jason J. Ong

**Affiliations:** ^1^ Faculty of Medicine and Health University of Sydney Sydney New South Wales Australia; ^2^ Faculty of Medicine, Dentistry and Health Sciences University of Melbourne Melbourne Victoria Australia; ^3^ Central Clinical School Monash University Melbourne Victoria Australia; ^4^ Melbourne Sexual Health Centre The Alfred Hospital Melbourne Victoria Australia; ^5^ Centre for Social Impact University of New South Wales Sydney New South Wales Australia; ^6^ Monash Outcomes Research and Health Economics Monash University Melbourne Victoria Australia; ^7^ Real World Data Enabling Platform Roche Products Ltd Welwyn Garden City UK

**Keywords:** HIV, quality of life, systematic review, people living with HIV, generic instrument, HIV‐specific instrument, PROSPERO Number: CRD42021240815

## Abstract

**Introduction:**

Due to the effectiveness of combined antiretroviral therapy and its growing availability worldwide, most people living with HIV (PLHIV) have a near‐normal life expectancy. However, PLHIV continue to face various health and social challenges that severely impact their health‐related quality‐of‐life (HRQoL). The UNAIDS Global AIDS Strategy discusses the need to optimize quality‐of‐life, but no guidance was given regarding which instruments were appropriate measures of HRQoL. This study aimed to review and assess the use of HRQoL instruments for PLHIV.

**Methods:**

We conducted a global systematic review and meta‐analysis, searching five databases for studies published between January 2010 and February 2021 that assessed HRQoL among PLHIV aged 16 years and over. Multivariable regression analyses were performed to identify factors associated with the choice of HRQoL instruments. We examined the domains covered by each instrument. Random‐effects meta‐analysis was conducted to explore the average completion rates of HRQoL instruments.

**Results and discussion:**

From 714 publications, we identified 65 different HRQoL instruments. The most commonly used instruments were the World Health Organization Quality‐of‐Life‐ HIV Bref (WHOQOL‐HIV BREF)—19%, Medical Outcome Survey‐HIV (MOS‐HIV)—17%, Short Form‐36 (SF‐36)—12%, European Quality‐of‐Life Instrument‐5 Dimension (EQ‐5D)—10%, World Health Organization Quality‐of‐Life Bref (WHOQOL BREF)—8%, Short Form‐12 (SF‐12)—7% and HIV/AIDS Targeted Quality‐of‐Life (HAT‐QOL)—6%. There were greater odds of using HIV‐specific instruments for middle‐ and low‐income countries (than high‐income countries), studies in the Americas and Europe (than Africa) and target population of PLHIV only (than both PLHIV and people without HIV). Domains unique to the HIV‐specific instruments were worries about death, stigma and HIV disclosure. There were no significant differences in completion rates between different HRQoL instruments. The overall pooled completion rate was 95.9% (95% CI: 94.7−97.0, *I* ^2^ = 99.2%, *p* < 0.01); some heterogeneity was explained by country‐income level and study type.

**Conclusions:**

A wide range of instruments have been used to assess HRQoL in PLHIV, and the choice of instrument might be based on their different characteristics and reason for application. Although completion rates were high, future studies should explore the feasibility of implementing these instruments and the appropriateness of domains covered by each instrument.

## 1 INTRODUCTION

The human immunodeficiency virus (HIV) affects approximately 38 million people globally [1, 2]. The Joint United Nations Programme on HIV/AIDS (UNAIDS) Global AIDS Strategy 2021–2026 aims to end the epidemic as a public health threat by 2030 [3]. Beyond the current response framework that focuses on testing, treatment and viral suppression, the new strategy adopts a more holistic, person‐centred approach to end inequalities and promote wellbeing. While viral suppression remains an important target, it should not be the only primary endpoint of the HIV care cascade. Many people living with HIV (PLHIV), despite being virologically suppressed, continue to experience significantly poorer health‐related quality‐of‐life (HRQoL) than the general population [4].

To continue to advocate for improved quality‐of‐life for PLHIV, we must accurately measure HRQoL. HRQoL is a multidimensional construct consisting of factors, such as physical, cognitive, emotional and social functioning. It falls under the broad banner of patient‐reported outcomes (PROs) [5]. A PRO is defined as a report of health status originating directly from a patient without secondary interpretation by a clinician [6]. In the post‐antiretroviral therapy era, many new PRO instruments—both generic and HIV‐specific—have been created to quantify HRQoL [7]. Generic PRO instruments allow comparisons across different diseases but may not adequately capture quality‐of‐life issues relevant to PLHIV, such as HIV‐related stigma or specific side effects related to antiretrovirals. On the other hand, condition‐specific PRO instruments are designed to be responsive to disease‐related changes but face challenges of comparability with other diseases [8].

Although many instruments exist, there are no recommendations regarding which instrument to use for PLHIV in research and clinical settings [9]. Furthermore, consistency on the specific dimensions that comprise HRQoL is lacking. To date, the scientific literature specifically comparing the different PRO measures and domains vital to HRQoL assessment of PLHIV is sparse. Knowledge about HRQoL has been derived mainly from experimental clinical drug trials examining HRQoL as a secondary measure, or studies exploring the impact of HIV on the HRQoL of PLHIV [10, 11, 12, 13].

Hence, this study aimed to review and critically appraise the use of the most common HRQoL instruments for PLHIV in research settings. Specifically, we provide an overview of which instruments are currently used and explore the association between the choice of generic or HIV‐specific instruments and explanatory factors, such as population, country income level, world region, settings, domains and feasibility. We also summarized the different domains included in the individual HRQoL instruments. Moreover, we examined the completion rates of HRQoL instruments and factors associated with higher completion rates. Accounting for past trends, this study seeks to add to the body of literature surrounding the instruments available and appropriate for assessing HRQoL among PLHIV.

## 2 METHODS

### 2.1 Search strategy and selection criteria

We followed the recommendations in the *Cochrane Handbook for Systematic Reviews* [14] and the PRISMA (The Preferred *Reporting* Items for Systematic reviews and Meta‐Analyses) reporting guidelines [15]. A comprehensive electronic literature search of five databases (Ovid Medline, Embase, PsycINFO, CINAHL and EconLit) was conducted on 28 February 2021. The search strategy was built around overarching terms, including “HIV,” “quality‐of‐life” and “patient‐reported outcomes,” and was adapted for each database (refer to Supplementary Material 1 [detailed search strategy]). Studies published from January 2010 to February 2021 were included. Additionally, a supplementary manual search of references cited by the included studies was completed to locate relevant papers missed by the search strategy. Studies were included if they used at least one instrument to measure HRQoL among adults (age 16 years and over) living with HIV. Papers that reported on HRQoL measures in children or measured HRQoL of PLHIV qualitatively or only focused on a narrow aspect of HRQoL (e.g. lipodystrophy) were excluded. Editorial letters without primary data, reviews, conference abstracts, protocol papers and non‐English language papers were excluded. At least two reviewers (YZ, CH and EH) independently screened the titles and abstracts, and another reviewer (JO) resolved any discrepancy. Given the large number of relevant full texts, three reviewers (YZ, CH and EH) extracted data from full texts, and a random 10% of full texts were checked by a fourth reviewer (JO). Data collected from each study included the study design, objective, population characteristics, PRO instrument administered, key domains addressed, reason for instrument choice, study setting and PRO‐related completion rate to assess instrument feasibility. The review was registered in PROSPERO (CRD42021240815) and completed on 21 September 2021.

### 2.2 Data analysis

Statistical analyses were conducted in R [16]. We used a Poisson regression model to determine any increasing trend in HRQoL‐related research and if it was statistically significant. Some papers utilized more than one instrument; these were counted separately. For instance, if a study used MOS‐HIV and SF‐36, we counted these as two independent instruments. The top HRQoL instruments were identified based on usage frequency, and a proportion trend test was performed to identify statistical significance over time. Logistic regression was conducted to identify factors associated with using an HIV‐specific instrument (compared to generic).

The key domains of each of the top seven HRQoL instruments were extracted and summarized. The names of the domains were taken from the wording in each HRQoL instrument questionnaire and were thematically analysed inductively. Domains were grouped if they had shared similarities. If an instrument had ≥1 item or question on mobility, it was judged to include a mobility domain.

To calculate the pooled proportions of completion of the HRQoL instrument, we used the function *metaprop* in STATA version 17 [17]. Statistical heterogeneity between studies was assessed with the *I* ^2^ statistic. Random‐effects meta‐regression models were conducted to explore study‐level factors (country income level, region of the world, study design, HRQoL instrument, type of instrument and number of items in the instrument) to explain the heterogeneity observed. We created funnel plots and used Egger's test to evaluate for small‐study effects. We used a non‐parametric “trim‐and‐fill” method, which imputes potentially missing studies to account for publication bias [18].

### 2.3 Role of funding source

The funder of the study had no role in study design, data collection, data analysis, data interpretation or writing of the report.

## 3 RESULTS AND DISCUSSION

**Figure 1:**
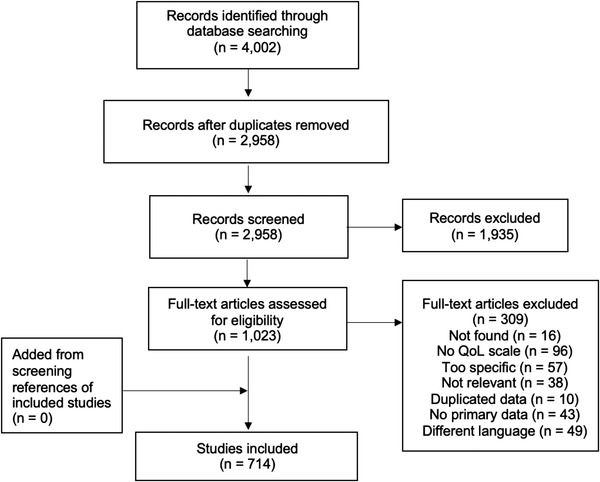
PRISMA flow diagram. This depicts the paper selection process.

The PRISMA flowchart is presented in Figure 1. A total of 714 publications met all the selection criteria and were included in the final review.

**Figure 2:**
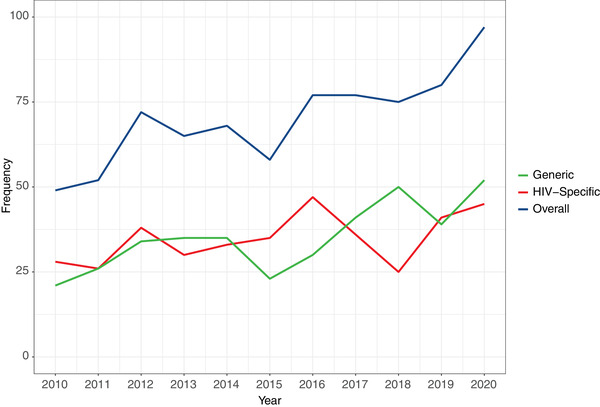
The number of HRQoL instruments used over time.

Across the 714 publications from 2010 to 2021, HRQoL instruments were used 770 times (Table 1). The number of publications increased over time (*p* < 0.001, Figure 2). The overall proportion of HIV‐specific (red line) and generic (green line) HRQoL instruments was 50.1% and 49.9%, respectively, with a consistent rising trend in research use over time (HIV‐specific: *p* < 0.001; generic: *p* < 0.001).

**Table 1:** Characteristics of studies utilizing HRQoL instruments (*N* = 770)

	*n*	Percentage (%)
Type		
Generic	386	50.1
HIV‐specific	384	49.9
Country income level		
High	344	44.7
Upper‐middle	232	30.0
Lower‐middle	119	15.4
Low	63	8.2
Mix	13	1.7
World region		
African	164	21.3
Americas	248	32.2
Eastern Mediterranean	22	2.9
European	131	17.0
South‐East Asian	73	9.5
Western Pacific	108	14.0
Mix	24	3.1
Study design		
RCT	110	14.3
Observational	505	65.6
Cohort	138	17.9
Economic evaluation	17	2.2
Study setting		
Hospital	405	52.6
Community/GP	219	28.4
NGO/peer‐led	32	4.2
Others	84	10.9
Unclear	30	3.9
Target population		
PLHIV versus non‐PLHIV	78	10.1
PLHIV only	692	89.9
Sub‐population characteristics		
Women	68	8.8
MSM	28	3.6
Mixed population (except women and MSM)	674	87.5

**Figure 3:**
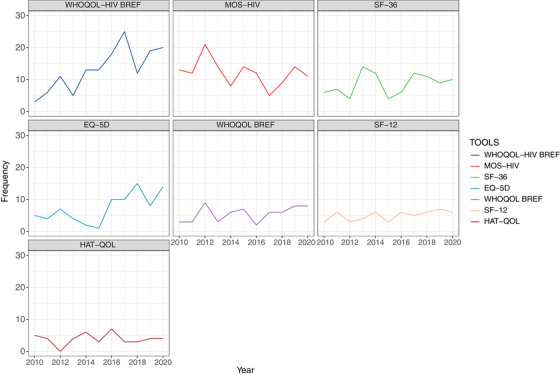
Frequency distribution of the most commonly used HRQoL instrument.

We identified 65 unique HRQoL instruments. The most frequently used instruments were World Health Organization Quality‐of‐life‐HIV Bref (WHOQOL‐HIV BREF)—19%, Medical Outcome Survey‐HIV (MOS‐HIV)—17%, Short Form‐36 (SF‐36)—12%, European Quality‐of‐life Instrument–5 Dimension (EQ‐5D‐3L or EQ‐5D‐5L)—10%, World Health Organization Quality‐of‐life Bref (WHOQOL BREF)—8%, Short Form‐12 (SF‐12)—7% and HIV/AIDS Targeted Quality‐of‐life (HAT‐QOL)—6%. Only the top seven HRQoL instruments were analysed in more detail in Figure 3, as the eighth instrument accounted for less than 3% of the total proportion. Supplementary Material 2 provides a detailed description of the most commonly used HRQoL instruments. The frequency of WHOQOL‐HIV BREF use reflects a clear increasing trend (*p* = 0.004), while the second most widely used instrument, MOS‐HIV, showed a declining trend in utilization (*p* = 0.004). The EQ‐5D‐3L or EQ‐5D‐5L, a generic HRQoL instrument, also had a statistically significant upward trajectory (*p* = 0.02). The frequency distribution of the other HRQoL instruments was fairly constant over time.

Multivariable logistic regression was conducted on studies that used an HIV‐specific instrument (compared to a generic instrument) as the outcome and country income level, world region, setting, study design, sub‐population characteristics and the target population as explanatory variables (Table 2). There was a significant relationship between country income level, study design, sub‐population characteristics and target population with the outcome of interest. There were greater odds of using HIV‐specific instruments for middle (OR: 3.53 [95% confidence interval [CI]: 2.10−5.95], *p* < 0.001; 3.96 [95% CI: 1.93−8.15], *p* = 0.002) and low‐income (OR: 11.57 [95% CI: 4.61−29.04], *p* < 0.001) countries compared to high‐income countries. HIV‐specific instrument, such as the WHOQOL‐HIV BREF, was more utilized in lower‐middle (27.8%) and low‐income (22.4%) countries compared to high‐income (7.8%) countries (Table S1). One plausible explanation is the WHO's continued effort in improving HIV programs in low‐to‐middle income countries that have a disproportionately high prevalence of HIV [19]. While the WHOQOL‐HIV is known to be sensitive to CD4 count and disease stage, it has been shown to adequately address HIV‐specific health concerns [20, 21, 22, 23, 24]. Another HIV‐specific instrument, the MOS‐HIV, was predominantly being used in low‐income countries (38.1%). This could be attributed to the instrument's well‐documented ceiling effects on physical function, role function, social function, cognitive function, pain and health transition subscales in PLHIV from high‐income countries [25]. Although studies in the Americas (OR: 2.02 [95% CI: 1.04−3.93], *p* = 0.039) and Europe (OR: 2.16 [95% CI: 1.01−4.63], *p* = 0.048) were more likely to use an HIV‐specific instrument than studies in Africa, it was possibly because in these regions that there was a higher proportion of studies focusing exclusively on PLHIV only (70.5% in Africa vs. 88.3% in Americas and 87.7% in Europe). This is consistent with our observation that studies examining just PLHIV (OR: 3.35 [95% CI: 1.80−5.95], *p* < 0.001) had a higher likelihood of employing an HIV‐specific instrument than studies examining both PLHIV and people without HIV. Particularly in clinical trial settings, HIV‐specific instruments may be more favourable to detect a clinically significant difference in outcome between the treatment and control groups. On the other hand, generic instruments, such as the SF‐36 (23.1%) and EQ‐5D (15.4%), were more frequently used in comparison studies (PLHIV vs. non‐PLHIV). Mixed populations (OR: 2.20 [95% CI: 1.25−3.88], *p* = 0.006) had greater odds of using an HIV‐specific instrument compared to women only. Table S1 provides further details of the HRQoL instruments by country income level, world region, setting, study design, sub‐population characteristics and the target population.

**Table 2:** Multivariable logistic regression of factors associated with using an HIV‐specific instrument compared with a generic health‐related quality‐of‐life instrument

Variables		Crude OR (95% CI)	*p*‐Value	Adjusted OR (95% CI)	*p*‐Value
Country income level	High	Reference		Reference	
	Upper‐middle	1.96 (1.40−2.75)	<0.001	3.53 (2·10−5.95)	<0.001
	Lower‐middle	1.93 (1.27−2.94)	0.002	3.96 (1·93−8.15)	0.002
	Low	4.95 (2.67−9.20)	<0.001	11.57 (4·61−29.04)	<0.001
	Mix	1.33 (0.44−4.03)	0.62	1.04 (0.24−4.57)	0.95
World region	African	Reference		Reference	
	Americas	0.59 (0.39−0.88)	0.009	2.02 (1.04−3.93)	0.039
	Eastern Mediterranean	0.58 (0.24−1.41)	0.23	0.64 (0.24−1.71)	0.38
	European	0.55 (0.34−0.87)	0.011	2.16 (1.01−4.63)	0.048
	South‐East Asian	0.79 (0.45−1.38)	0.41	0.95 (0.51−1.80)	0.89
	Western Pacific	0.72 (0.44−1.17)	0.18	1.06 (0.57−1.95)	0.86
	Mix	0.58 (0.25−1.38)	0.22	2.34 (0.66−8.30)	0.19
Study setting	Hospital	Reference		Reference	
	Community/GP	0.83 (0.60−1.16)	0.28	0.80 (0.56−1.15)	0.23
	NGO/peer‐led	0.84 (0.41−1.74)	0.64	1.07 (0.48−2.35)	0.87
	Others	0.96 (0.60−1.53)	0.85	1.14 (0.69−1.90)	0.61
	Unclear	1.43 (0.67−3.06)	0.35	1.76 (0.78−3.97)	0.17
Study design	RCT	Reference		Reference	
	Observational	0.88 (0.58−1.33)	0.53	0.79 (0.51−1.23)	0.30
	Cohort	0.74 (0.45−1.23)	0.24	0.78 (0.45−1.34)	0.37
	Economic evaluation	0.00 (0.00−Inf)	0.96	0.00 (0.00−Inf)	0.98
Target population	PLHIV versus non‐PLHIV	Reference		Reference	
	PLHIV only	3.47 (2.02−5.94)	<0.001	3.35 (1.80−5.95)	<0.001
Sub‐population characteristics	Women	Reference		Reference	
	MSM	1.47 (0.60−3.61)	0.40	2.05 (0.77−5.44)	0.15
	Mixed population (except women and MSM)	2.10 (1.24−2.44)	0.006	2.20 (1.25−3.88)	0.006

Note: *p*‐value<0.05: association is statistically significant. Likelihood ratio test compared the use of an HIV‐specific instrument (coded as 1) compared to a generic instrument (coded as 0).

Abbreviations: GP, general practitioner; MSM, men who have sex with men; NGO, non‐government organization; OR, odds‐ratio; PLHIV, people living with HIV; RCT, randomized controlled trial; 95% CI, confidence interval.

Table 3 summarizes the domains covered by the most commonly used HRQoL instruments. Table S2 also provides details on how items were categorized into their respective domains. All the instruments covered items on mobility, usual activities, pain and negative feelings (distress, anxiety/depression and nervousness). Most instruments (6/7; 86%) covered positive feelings (enjoyment, calm, satisfaction and happiness), energy and social items. More than half (4/7; 57%) covered general health items, while only three (43%) covered cognition, medical treatment, acceptance by others/self, sexual health, financial security, and access and quality of health services. Less common items (2/7; 29%) were bodily appearance (WHOQOL‐HIV BREF and WHOQOL BREF), worries about death (HAT‐QOL and WHOQOL‐HIV BREF), spirituality/religion (WHOQOL‐HIV BREF and WHOQOL BREF) and environment/safety (WHOQOL‐HIV BREF and WHOQOL BREF). Items that were rare were HIV disclosure (HAT‐QOL) and blame for HIV status/stigma (WHOQOL‐HIV BREF). The items that were unique to the HIV‐specific instruments compared to generic were worries about death, stigma and HIV disclosure.

**Table 3:** Domains captured by the most commonly used HRQoL instruments

Name of instrument	General health	Mobility	Usual activities	Pain	Negative feelings (anxiety/depression)	Positive feelings	Energy and vitality	Cognitive	Bodily appearance, self‐esteem	Medical treatment	Acceptance and support by others or self	HIV disclosure	Relationships, social	Sex life	Safety/physical environment	Financial security	Access and quality of info/health services	Spirituality/religion	Blame for HIV status/stigma	Death worry
Generic instruments																				
EQ‐5D‐3L or EQ‐5D‐5L		X	X	X	X															
SF‐36	X	X	X	X	X	X	X						X							
SF‐12	X	X	X	X	X	X	X						X							
WHOQOL BREF		X	X	X	X	X	X	X	X	X	X		X	X	X	X	X	X		
HIV‐specific instruments																				
WHOQOL‐HIV BREF		X	X	X	X	X	X	X	X	X	X		X	X	X	X	X	X	X	X
HAT‐QOL	X	X	X	X	X	X	X			X	X	X	X	X		X	X			X
MOS‐HIV	X	X	X	X	X	X	X	X					x							

A total of 233 studies provided information about completion rates. We found a pooled completion rate of 95.9% (95% CI: 94.7−97.0, *I* ^2^ = 99.2%, *p* < 0.01). Table S3 provides completion rates according to subgroups of country‐income level, region of the world, study design, HRQoL instrument, type of instrument and number of items in the instrument. The completion rate of HIV‐specific instruments (97.3%) was higher than generic ones (94.6%), while instruments with 24−32 items had the highest completion rate of 97.4%. Generic instruments may be more time‐consuming to complete due to their vast scope, particularly for respondents for whom the concepts may be less relevant or difficult to relate [26]. The completion percentage decreased as the number of items in the questionnaire increased (32−35 items: 97.0%, 36−120 items: 95.4%). It is possible that the length of the questionnaire is associated with respondent fatigue [27], and hence, a lower completion rate was observed for questionnaire with more questions. Furthermore, longer questionnaires may be better tolerated in research settings than in clinical practice. Cross‐sectional studies and randomized controlled trials both reported high completion rates at 97.1%; however, cohort studies indicated significantly lower completion rates (88.7%). Cohort studies generally have broader inclusion criteria compared to randomized controlled trials and may be at risk of attrition effect [28]. The meta‐regression (Table S4) showed that there were significantly higher completion rates for studies from lower‐middle‐income countries (compared to high‐income) and cross‐sectional and randomized controlled trials (compared to cohort studies). Among all the variables, country income level and study design explained most of the variability in the completion rate (adjusted *R* ^2^ = 10.5% and 7.5%, respectively). We did not find any significant association between completion rates and the world region, specific instrument, generic versus HIV‐specific instruments or the number of items in the instrument. Figure S1 shows potential for publication bias, where studies with a higher completion rate are more likely to be published. When we addressed this by imputing potential missing studies (Figure S2), we found a corrected pooled completion rate of 90.6% (95% CI: 89.1−92.1).

As we shift towards a new paradigm in managing HIV infection as a chronic illness, more emphasis has been placed on PROs. Given the myriad of PRO‐based HRQoL instruments and diverse domains to measure HRQoL, this systematic review and meta‐analysis collated and assessed the use of PRO‐based HRQoL instruments for PLHIV. We add to the literature by presenting an overview of the current trend of HRQoL instruments among PLHIV and identified the most commonly used instruments in recent years––WHOQOL‐HIV BREF, MOS‐HIV, SF‐36, EQ‐5D, WHOQOL BREF, SF‐12 and HAT‐QOL. There were greater odds of using HIV‐specific instruments for middle‐ and low‐income countries (than high‐income countries), studies in the Americas and Europe (than Africa) and target population of PLHIV only (than both PLHIV and people without HIV). We illustrated the coverage of domains for these instruments and found that domains unique to the HIV‐specific instruments were worries about death, stigma and HIV disclosure. Completion rates were generally high at an overall pooled percentage of 95.9% and were associated with country income level and study design.

The increase in publications using HRQoL instruments among PLHIV may reflect increased attention on the importance of measuring HRQoL or reflect the secular trend of more publications over time. Nevertheless, we noted the rising popularity of WHOQOL‐HIV BREF and EQ‐5D‐3L/EQ‐5D‐5L and declining use of MOS‐HIV. The WHOQOL‐HIV BREF was developed by the World Health Organization to capture quality‐of‐life cross‐culturally in PLHIV and is available in more than 20 languages, hence, its popularity may be explained by its extensive applicability across many cultural contexts [29]. WHO instruments are also one of the only HRQoL scales incorporating a spirituality domain. Spirituality has been demonstrated to contribute significantly to the health of patients afflicted with a severe terminal illness, such as HIV, and such factors may be increasingly important as clinicians shift towards a more holistic view of HIV care [30]. The EQ‐5D‐3L/EQ‐5D‐5L is a very time‐efficient instrument, requiring 1 minute on average to complete. This may be attractive to researchers measuring multiple clinical endpoints in clinical trials or relationships between multiple variables in observational studies. It is also a particularly helpful generic tool for epidemiological investigations since it enables the comparison of managements or populations regardless of the disease [31]. Furthermore, EQ‐5D‐3L/EQ‐5D‐5L scale scores can be converted to cost‐utility scores, which may be useful for studies with the aim of directing healthcare policy development and evaluating medical interventions [32]. MOS‐HIV is responsive to clinically relevant outcomes, including adverse events, increased symptoms, opportunistic infections and HIV‐related events [33, 34]. Psychometric testing of the MOS‐HIV has demonstrated ceiling effects and less efficacy in capturing health changes in PLHIV past a certain threshold of good health, which may contribute to decreased applicability alongside the increasing success of antiretroviral therapy (ART) in controlling viral load [25]. However, the changes in trends of WHOQOL‐HIV BREF, EQ‐5D‐3L/EQ‐5D‐5L and MOS‐HIV may be more likely due to differing preferences of clinicians and researchers than any limitations in the MOS‐HIV instrument.

Interestingly, we found an equal use of both generic and HIV‐specific instruments, with both types equally rising over time. However, we found several factors associated with the choice of the instrument. For example, studies that compared PLHIV to non‐PLHIV had greater odds of using generic instruments because generic instruments can capture the difference in HRQoL between people with and without the disease [35], making it suitable for comparison studies. The SF‐36 was the most frequently used generic instrument in comparison studies due to its versatility in a variety of contexts and populations, and the availability of representative normative population data for various nations allows for comparisons between study samples and general population data [36]. Moreover, it correlates well with disease severity showing good construct validity [37], and is responsive to changes in viral load [5], making it the most appropriate generic instrument for assessing HRQoL in PLHIV as affirmed by Colautti et al. [38]. Furthermore, there is more evidence for the SF‐36 in PLHIV than other generic measures (e.g. EQ‐5D) [5]. Health utility scores may be calculated using the SF‐36, enabling policymakers to evaluate the cost and quality‐adjusted life years gained across competing priorities for various diseases. Conversely, studies that focus only on PLHIV are more likely to use an HIV‐specific instrument (WHOQOL‐HIV BREF or MOS‐HIV), as they are typically clinical or research studies that involve an HIV‐specific intervention or measure symptoms that are directly addressed by the intervention. We also found that country‐income level and world region had a different likelihood of using HIV‐specific instruments than generic instruments even after adjusting for other factors like study design. There were greater odds of using HIV‐specific instruments for middle‐ and low‐income countries compared to high‐income countries. Studies in the Americas and Europe were more likely to use an HIV‐specific instrument than studies in Africa. The choice of generic versus HIV‐specific instruments also depends on which HRQOL domains are important to measure. Among the commonly used HRQoL instruments, only one instrument captured HIV disclosure (HAT‐QOL) and blame for HIV status or stigma (WHOQOL‐HIV BREF). However, stigma is a persistent feature of HIV infection [39] and stigmatized individuals are at a higher risk of suffering mental strain and a decline in their psychological wellbeing [40, 41]. A study on PLHIV in Spain concluded that both enacted and internalized stigmas are associated with reduced HRQoL [40, 42]. Given that stigma can severely impact PLHIV, HRQoL instruments that measure this (e.g. PROQOL and WHOQOL‐HIV BREF) should be preferred. In addition, instruments that can capture the impact of broader drivers of quality of life affecting PLHIV, such as financial insecurity and other environment factors, may be useful, especially in low‐income countries, where PLHIV face poverty, inadequate health and social infrastructure, and food insecurity. These data will better inform policymakers about the needs of PLHIV and facilitate a coordinated response to also address the broader social determinants of health, including poverty alleviation, sustainable food systems and infrastructure improvement. Although we found a very high completion rate for all instruments, with no obvious instrument completed more frequently than others, this could also be because of publication bias. We did not identify any data related to comparing the cost or resources needed for using each instrument, which may also affect the choice of instrument.

Our study should be read in light of some limitations. We were limited by the data available from published studies. PROs which were published earliest are more likely to have been used in studies and published literature, presenting a bias in the number of studies towards older PROs. It is likely that different or more recently developed quality‐of‐life instruments may be used programmatically with no or fewer academic publications regarding their use in practice. In evaluating the completion rates of the instruments, studies may utilize different criteria for the definition of a complete survey. There is also evidence of publication bias, indicating that studies with lower completion rates were less likely to be published.

## 4 CONCLUSIONS

This systematic review and meta‐analysis provide an overview of the current HRQoL instruments used for PLHIV. The priorities and focus of health services research may evolve as services strive to meet the quality‐of‐life needs for PLHIV and so increase the demand for measuring HRQoL. Understanding the current use of the most common HRQoL instruments, the dimensions being measured, the contexts of their use and how usage can change over time may guide the selection and use of HRQoL instruments for policy, clinical and research settings.

## COMPETING INTERESTS

ROA is an employee of Roche Products Ltd. The views expressed in this article are his own and do not represent that of his employers. The remaining authors declare that the research was conducted in the absence of any commercial or financial relationships that could be construed as a potential competing interest.

## AUTHORS’ CONTRIBUTIONS

JJO conceived the idea for this paper. YZ, CH and ESGH did the screening and data extraction. YZ and JJO conducted the statistical analysis. All authors had full access to all the data in the study, contributed to the interpretation and subsequent edits of the manuscript, and had final responsibility for the decision to submit for publication.

## FUNDING

JJO is funded by the Australian National Health and Medical Research Council (NHMRC)––Investigator Grant 1193955.

## Supporting information


**Supplementary Material 1**: Detailed search strategy.

**Supplementary Material 2**: Summary of health‐related quality‐of‐life instruments.

**Figure S1**: Funnel plot for included studies.

**Figure S2**: Funnel plot for included and imputed studies.

**Table S1**: Distribution of the most commonly used HRQoL instruments.

**Table S2**: Coding for the categorisation of the items.

**Table S3**: Sub‐analysis of pooled completion rates of HRQoL instruments.

**Table S4**: Meta‐regression results of completion rate of HRQoL instruments.

## Data Availability

The authors confirm that the data supporting the findings of this study are available within the article and its Supplementary Materials.
