# Supplementary material for: Use of quality‐of‐life instruments for people living with HIV: a global systematic review and meta‐analysis
Source: J Int AIDS Soc. 2022 Apr 9;25(4):e25902. doi: 10.1002/jia2.25902 (PMC8994483; doi:10.1002/jia2.25902)
Supplement: Supplementary file 1 — Supplementary Material 1: Detailed search strategy. [file JIA2-25-e25902-s001.docx]

## Supplementary 1. Detailed search strategy

Ovid Medline + Embase + PsycINFO

| **#** | **Query** |
| --- | --- |
| 1. | (HIV or “Human Immunodeficiency Virus” or antiretroviral*).mp. |
| 2. | ("quality of life" or “life quality” or QoL or PROM or “patient reported outcome).mp. |
| 3. | (measure* or inventor* or scale or questionnaire* or self-report* or assessment* or survey* or tool* or indicator* or instrument*).mp. |
| 4 | 1 and 2 and 3 |
| 5 | Limit 4 to yr= “2010 – 2020” |

Ebsco CINAHL Complete + EconLit

| **#** | **Query** |
| --- | --- |
| 1. | TX (HIV or “Human Immunodeficiency Virus” or antiretroviral*) |
| 2. | TX ("quality of life" or “life quality” or QoL or PROM or “patient reported outcome) |
| 3. | TX (measure* or inventor* or scale or questionnaire* or self-report* or assessment* or survey* or tool* or indicator* or instrument*) |
| 4 | 1 and 2 and 3 |
| 5 | Limit 4 to yr= “2010 – 2020” |
