# Supplementary material for: Use of quality‐of‐life instruments for people living with HIV: a global systematic review and meta‐analysis
Source: J Int AIDS Soc. 2022 Apr 9;25(4):e25902. doi: 10.1002/jia2.25902 (PMC8994483; doi:10.1002/jia2.25902)
Supplement: Supplementary file 2 — Supplementary Material 2: Summary of health‐related quality‐of‐life instruments. [file JIA2-25-e25902-s003.docx]

**Supplementary 2. Summary of health-related quality-of-life instruments**

*Generic QoL instruments*

**EQ-5D**

The EQ-5D is a generic 5-item quality of life (QoL) instrument encompassing five domains: mobility, self-care, usual activities, pain/discomfort, anxiety/depression. This instrument is often accompanied by a visual analogue scale measuring overall health. There are two versions of this instrument; the original EQ-5D-3L, where each of the five domains is rated on a 3-point-scale and a newer EQ-5D-5L instrument, where domains are rated on a 5-point scale [1]. The original EQ-5D instrument was developed for use in clinical trials and observational studies, either standalone or simultaneously with other patient-reported outcomes [2]. EQ5D takes approximately 1 minute to complete [3, 4]. Both the EQ-5D-3L and EQ-5D-5L have relatively high acceptability and completion (>90%) for people living with HIV (PLHIV) in both hospital, clinic and outpatient settings, possibly due to its time efficiency and convenience [5-7]. General population study participants from high-income European countries have reported the EQ-5D-5L to have well-formulated and specific questions, clear scales differentiating severity of disease and broad coverage of different dimensions of health, indicating acceptable face validity and content validity in high-income countries [8]. EQ-5D scores can be converted to health utility values, allowing cost-utility analysis which may be useful for the economic evaluation of medical interventions [2, 9]. However, there has been conflicting evidence on the sensitivity of EQ-5D to detecting changes in PLHIV. Some studies have found EQ-5D to be responsive to ART treatment, with the ability to detect patients’ clinical stage of ART, as well as alcohol and drug use [4]. However, EQ-5D (especially the EQ-5D-3L) has reported high ceiling effects and low responsiveness to HRQoL changes unrelated to health utility when utilised in the context of an international RCT targeted toward PLHIV [2, 10].

**SF-36**

The Short Form-36 (SF-36) is a generic QoL instrument encompassing eight domains: physical function (PF), social function (SF), role limitations due to physical health (or ‘role physical’) (RP), role limitations due to emotional problems (or ‘role emotional’) (RE), mental health (MH), vitality (VT), bodily pain (BP), and general health (GH), which contribute to physical component summary (PCS) and mental component summary (MCS) scores. This instrument takes approximately 5-10 minutes to complete. Developers of SF36 prioritised content validity and selected items, which could accurately measure medically and socially relevant differences in health status reflective of various medical and psychiatric conditions [11]. There are two versions of the SF-36: the original SF-36V1 represented on a 0-100 scale where higher ratings represented better health and a revised SF-36V2, which can be reported as either a percentage or “norm-based” T-score.[12] Health domains that may be relevant to PLHIV, such as health distress, sexual functioning, cognitive functioning and sleep quality, were omitted from the final SF-36 [11]. However, in general, SF-36 is responsive to the severity of ill health, as well as specific conditions such as depression [13, 14]. SF-36 has been used in various contexts and populations with representative normative population data compiled for different nations, allowing for comparison between study samples and general population data [15]. This measure has shown good test-retest reliability in PLHIV in psychometric testing as well as cross-sectional studies in middle- and high-income countries [13, 16-18]. SF-36 has demonstrated high completion rates across a range of settings (i.e., hospital, clinic) and studies (i.e., RCT, cohort studies, cross-sectional studies) [16-18].

**SF-12**

The SF-12 is a shorter adaptation of SF-36 (described above). The SF-12 is a 12-item generic QoL instrument, encompassing eight domains: physical function (PF), social function (SF), role limitations due to physical health (or ‘role physical’) (RP), role limitations due to emotional problems (or ‘role emotional’) (RE), mental health (MH), vitality (VT), bodily pain (BP), and general health (GH), which contribute to physical health summary (PHS) and mental health summary (MHS) scores. The SF-12 can be completed in 2 minutes or less and is able to provide a snapshot of patients’ HRQoL [19, 20]. The SF-12 is an abbreviated form of the SF-36 instrument. The SF-12 is less comprehensive at capturing information about health status and outcomes than the SF-36; however, in studies aiming to investigate overall physical and mental health in HIV patients, SF-12 may be sufficient in capturing the relevant information in a large sample size [19, 21]. SF-12 is responsive to disease severity (measured by CD4+ cell count and viral RNA levels) [21]. Psychometric studies investigating the SF-12 in European, US and Tunisian populations did not detect any floor or ceiling effects [19, 22, 23].

**WHOQOL BREF**

The WHOQOL BREF is a 26-item generic QoL instrument encompassing four domains: physical health, psychological health, social relationships and environment and two general items measuring overall quality of life and general health. Items are rated on a 5-point Likert scale and summated to give an overall QoL rating, with higher ratings indicating better QoL. This HRQoL instrument is an abbreviated version of the WHOQOL-100. The instrument takes approximately 6 minutes to complete and may be preferred in busy clinic and hospital settings. The general but comprehensive nature of WHOQOL BREF has allowed for comparison between different disease populations, countries and cultures as well as with a general population [24, 25]. WHOQOL BREF items concentrate more on the relationship between individuals and their everyday social contexts rather than isolated individual feelings and behaviours, allowing for social determinants of health to be investigated, assisting health providers and policy developers in investigating useful interventions at a community program level [26]. However, the content of the WHOQOL BREF may not be most aligned with aspects of QoL most relevant to PLHIV, as important items such as HIV-specific symptoms and discrimination experienced by PLHIV are not measured by the WHOQOL BREF [24, 27]. This instrument has also been found to be responsive to changes in HRQoL over time [28]. It has been reported that WHOQOL BREF is easy for patients to complete [25] and the instrument has achieved high completion rates >80% across a range of settings (i.e., hospital, clinic) and studies (i.e., RCT, cohort studies, cross-sectional studies) [24, 29, 30].

*HIV-specific QoL instruments*

**MOS-HIV**

The Medical Outcomes Study HIV (MOS-HIV) is a 35-item HIV-specific QoL instrument encompassing 11 domains: quality of life QL), general health perceptions (GH), physical function (PF), role function (RF), social function (SF), cognitive function (CF), pain (PN), mental health (MH), energy (EN), health distress (HD) and health transition (HT). Scores obtained in each domain contribute towards a physical health summary (PHS) and mental health summary (MHS) score. PF, PN, and RF scale scores contribute most strongly to PHS. MH, HD, QL, and CF scales contribute most strongly to MHS. EN, OH and SF contribute to both factors. MOS-HIV subscales contribute towards a summated 0-100 scale, with higher ratings indicating better health. This instrument generally takes 5 minutes to complete [31]. MOS-HIV has demonstrated high sensitivity to clinically relevant changes in a broad range of HIV+ populations, including disease progression and exacerbation, ART treatment, ART adverse events and development of opportunistic infections [31-34]. MOS-HIV has also shown good known group validity and differentiate between patients in different clinical disease stages [32, 34]. MOS-HIV has been culturally adapted and translated into a diverse range of languages [34]. MOS-HIV is an acceptable measure of patient outcomes in clinical trials, cohort and cross-sectional studies, with relatively high completion rates in various settings, including hospitals, clinics and community-based organisations [33, 35-38]. MOS-HIV contains both generic and HIV specific items, allowing comparison of HRQoL in PLHIV with healthy populations [39]. The health transition subscale in MOS-HIV captures how perceived changes in health will impact other MOS-HIV domain scores [39]. However, psychometric testing of MOS-HIV in a general high-income country among PLHIV has revealed ceiling effects in PF, RF, SF, CF, PN and HT subscales, indicating that MOS-HIV may not be effective in capturing health changes in HIV+ individuals past a certain threshold of good health.[39]

**WHOQOL-HIV-BREF**

The WHOQOL-HIV BREF is a 31-item HIV-specific QoL instrument encompassing six domains: physical, psychological, level of independence, social relations, environment and spiritual. This instrument was an abbreviated version of the 120-item WHOQOL-HIV instrument, developed by the World Health Organisation to cross-culturally capture QoL in PLHIV [40]. The 31 items are rated on a 5-point Likert scale and summated to give an overall QoL rating with higher ratings indicating better QoL. The inclusion of a spiritual domain is unique to WHO developed QoL instruments [41]. Spirituality has been shown to contribute significantly to the health of patients afflicted with a terminal or severe illness such as HIV and hence may be relevant to HIV+ populations [41]. The multidimensional nature of WHOQOL-HIV BREF subscales has been reported to address HIV-specific health concerns adequately [42]. WHOQOL-HIV BREF is sensitive to CD4 count and the clinical stage of the disease [42-46]. Floor and ceiling effects were identified in the WHOQOL-HIV instrument when psychometrically tested in European HIV+ populations [47]. The WHOQOL-HIV BREF has been translated into many different languages; it has been used cross-culturally across a range of settings (i.e., hospital, clinic) and studies (I.e., RCT, cohort studies, cross-sectional studies), achieving high completion rates in most studies [46, 48].

**HAT-QOL**

The HAT-QOL is a 42-item HIV-specific QoL instrument encompassing nine domains: overall function, life satisfaction, health worries, financial worries, medication worries, HIV mastery, disclosure worries, provider trust and sexual function. Items are rated on a Likert scale describing the frequency with which HIV patients experienced events pertaining to the nine domains across the previous four weeks: all the time, a lot of the time, some of the time, a little of the time, none of the time. Subscale scores are scaled onto a 0-100 scale, with higher scores indicating a greater QoL. There is also a shortened 34-item version of this instrument. HAT-QoL takes an average of 10-15 minutes to complete. The HAT-QoL instrument was constructed from personal suggestions of PLHIV; hence it is thought to contain items reflecting items pertinent to the QoL for PLHIV [49]. However certain domains such as sexual function may be inappropriate for more conservative populations such as some Sub-Saharan African populations. HAT-QoL is sensitive to changes in asymptomatic seropositive individuals at the healthier end of the spectrum. The provider trust domain has exhibited ceiling effects however other domains of HAT-QoL appear to have minimal ceiling effects [39]. HAT-QoL has been described as easily comprehensible with the ability to be applied to a range of research contexts, including cross-sectional, cohort and RCT studies in both hospital and clinical settings [49].

**References**

1. Jia YX, Cui FQ, Li L, Zhang DL, Zhang GM, Wang FZ, et al. Comparison between the EQ-5D-5L and the EQ-5D-3L in patients with hepatitis B. Qual Life Res. 2014;23(8):2355-63.

2. Wu A, Jacobson K, Frick K, Clark R, Revicki DA, Freedberg KA, et al. Validity and responsiveness of the EUROQOL as a measure of health-related quality of life in people enrolled in an AIDS clinical trial. Qual Life Res. 2002;11(3):273-82.

3. Wang X, Guo G, Zhou L, Zheng J, Liang X, Li Z, et al. Health-related quality of life in pregnant women living with HIV: a comparison of EQ-5D and SF-12. Health Qual Life Outcomes. 2017;15(1):158.

4. Tran B, Ohinmaa A, Nguyen L. Quality of life profile and psychometric properties of the EQ-5D-5L in HIV/AIDS patients. Health Qual Life Outcomes. 2012;10:132.

5. Brañas F, Sánchez-Conde M, Carli F, Menozzi M, Raimondi A, Milic J, et al. Sex difference in people aging with HIV. J Acquir Immune Defic Syndr. 2020;83(3):284-91.

6. Martin K, Naclerio F, Karsten B, Vera J. Physical activity and quality of life in people living with HIV. AIDS Care. 2019;31(5):589-98.

7. Tran B, Ohinmaa A, Nguyen L, Anh N, Nguyen T. Determinants of health-related quality of life in adults living with HIV in Vietnam. AIDS Care. 2011;23(10):1236-45.

8. Herdman M, Gudex C, Lloyd A, Janssen MF, Kind P, Parkin D, et al. Development and preliminary testing of the new five-leve version of EQ-5D (EQ-5D-5L). Qual Life Res. 2011;20(10):1727-36.

9. Dolan P. Modelling Valuations for EuroQOL Health States. Medical care. 1997;35:1095-108.

10. Johnson JA, Coons S. Comparison of the EQ-5D and SF-12 in an adult US sample. Qual Life Res. 1998;7:155-66.

11. Ware J, Sherbourne C. The MOS 36-item short-form health survey (SF-36). I. Conceptual framework and item selection. Med Care. 1992;30:473-83.

12. Hawthorne G, Osborne R, Taylor A, Sansoni J. The SF36 Version 2: critical analyses of population weights, scoring algorithms and population norms. Qual Life Res. 2007;16(4):661-73.

13. Brazier JE, Harper R, Jones NM, O'Cathain A, Thomas KJ, Usherwood T, et al. Validating the SF-36 health survey questionnaire: new outcome measure for primary care. BMJ. 1992;305(6846):160-4.

14. Valdelamar J, Lins-Kusterer L, Jesus S, Netto E, Brites C. Comparison of three health-related quality of life instruments to evaluate symptoms of depression in HIV patients in Brazil. J Clin Psychol Med Settings. 2020;27(4):643-50.

15. Shahriar J, Delate T, Hays R, Coons S. Commentary on using the SF36 or MOS-HIV in studies of persons with HIV disease. Health Qual Life Outcomes. 2003;1:25-7.

16. Sun W, Wu M, Qu P, Lu C, Wang L. Quality of life of people living with HIV/AIDS under the new epidemic characteristics in China and the associated factors. PLoS One. 2013;8(5):e64562.

17. Shrestha R, Copenhaver M, Bazazi A, Huedo-Medina T, Krishnan A. A moderated mediation model of HIV-related stigma, depression, and social support on health-related quality of life among incarcerated Malaysian men with HIV and opioid dependence. AIDS Behav. 2017;21(4):1-11.

18. Dalmida SG, Koenig HG, Holstad MM, MM W. The psychological well-being of people living with HIV/AIDS and the role of religious coping and social support. Int J Psychiatry Med. 2013;46(1):57-83.

19. Ware J, Kosinski MM, Keller S. A 12-Item short-form health survey: construction of scales and preliminary tests of reliability and validity. Med Care. 1996;34(3):220-33.

20. Chariyalertsak S, Wansom T, Kawichai S, Ruangyuttikarna C, Kemerer VF, Wu AW. Reliability and validity of Thai versions of the MOS-HIV and SF-12 quality of life questionnaires in people living with HIV/AIDS. Health Qual Life Outcomes. 2011;9:15.

21. Delate T, Coons S. The discriminative ability of the 12-item Short Form Health Survey (SF-12) in a sample of persons infected with HIV. Clin Ther. 2000;22:1112-20.

22. Younsi M. Health-related quality-of-life measures: evidence from Tunisian population using the SF-12 health survey. Value Health Reg Issues. 2015;7:54-66.

23. Gandek B, Ware J, Aaronson N, Apolone G, Bjorner J, Brazier J, et al. Cross-validation of item selection and scoring for the SF-12 health survey in nine countries: results from the IQOLA Project. J Clin Epidemiol. 1998;51(11):1171-8.

24. Wakawa IA, Said JM, Abba WM, Shehu S, Rabbebe IB, Beida O. The impact of comorbid clinical depression on the health-related quality of life of adults on highly active antiretroviral therapy in Maiduguri, northeastern Nigeria. Indian J Psychol Med. 2014;36(4):408-17.

25. Liping M, Peng X, Haijiang L, Lahong J, Fan L. Quality of life of people living with HIV/AIDS: a cross-sectional study in Zhejiang Province, China. PLoS One. 2015;10(8):e0135705.

26. Thompson H, Reisner S, Vankim N, Raymond H. Quality-of-life measurement: Assessing the WHOQOL-BREF scale in a sample of high-HIV-risk transgender women in San Francisco, California. Int J Transgend. 2015;16(1):36-48.

27. Fang C, Hsiung P, Yu C, Chen M, Wang J-D. Validation of the WHO quality of life instruments in patients with HIV infection. Qual Life Res. 2002;11(8):753-62.

28. Rongkavilit C, Wright K, Chen X, Naar-King S, Chuenyam T, Phanuphak P. HIV stigma, disclosure and psychosocial distress among Thai youth living with HIV. Int J STD AIDS. 2010;21(2):126-32.

29. Wang H, Zhou J, Huang L, Li X, Fennie KP, Williams AB. Effects of nurse-delivered home visits combined with telephone calls on medication adherence and quality of life in HIV-infected heroin users in Hunan of China. J Clin Nurs. 2010;19(3-4):380-8.

30. Rzeszutek M, Gruszczynska E. Consistency of health-related quality of life among people living with HIV: Latent state-trait analysis. Health Qual Life Outcomes. 2018;16(1):101.

31. Revicki D, Sorensen S, Wu A. Reliability and validity of physical and mental health summary scores from the Medical Outcomes Study HIV Health Survey. Med Care. 1998;36(2):126-37.

32. Revicki D, Wu A, Murray M. Change in clinical status and health utility outcomes in HIV-infected patients. Med Care. 1995;33:AS173-82.

33. Wu A, Hays R, Kelly S, Malitz F, Bozzette S. Applications of the Medical Outcomes Study health-related quality of life measures in HIV/AIDS. Qual Life Res. 1997;6:531-54.

34. Stasinopoulou P, Tzavara C, Dimitrakaki C, Georgiou O, Baraboutis I, Skoutelis A, et al. Reliability and validity of the Greek translation of the MOS-HIV health survey in HIV-infected individuals. Qual Life Res. 2010;19(2):199-205.

35. Xie F, Zheng H, Huang L, Yuan Z, Lu Y. Social capital associated with quality of life among people living with HIV/AIDS in Nanchang, China. Int J Environ Res Public Health. 2019;16(2):276.

36. McDonald C, Uy J HW, Wirtz V, Juethner S, Butcher D, McGrath D, et al. Clinical significance of hyperbilirubinemia among HIV-1-infected patients treated with atazanavir/ritonavir through 96 weeks in the CASTLE study. AIDS Patient Care STDS. 2012;26(5):259-64.

37. Huang Y, Luo D, Chen X, Zhang D, Huang Z, Xiao S. Role of psychosocial status in predicting health-related quality of life at 1-year follow-up among newly diagnosed people living with HIV. PLoS One. 2019;14(10):e0224322.

38. Maluccio J, Palermo T, Kadiyala S, Rawat R. Improving health-related quality of life among people living with HIV: results from an impact evaluation of a food assistance program in Uganda. PLoS One. 2015;10(8):e0135879.

39. Holmes W, Shea J. Two approaches to measuring quality of life in the HIV/AIDS population: HAT-QoL and MOS-HIV. Quality of life research : an international journal of quality of life aspects of treatment, care and rehabilitation. 1999;8:515-27.

40. Ndubuka N, Lim H, Ehlers V, van der Wal D. Health-related quality of life of patients on antiretroviral treatment in Botswana: A cross-sectional study. Palliative and Supportive Care. 2016;-1:1-9.

41. WHOQOL HIV Group. WHOQOL-HIV for quality of life assessment among people living with HIV and AIDS: results from the field test. AIDS Care. 2004;16(7):882-9.

42. Canavarro M, Pereira M. Factor structure and psychometric properties of the European Portuguese version of a questionnaire to assess quality of life in HIV-infected adults: The WHOQOL-HIV-Bref. AIDS care. 2012;24:799-807.

43. Bhowmik A, Ghugre P, Udipi S, Guha S. Nutritional status and quality of life of women with HIV/AIDS. Am J Infect Dis. 2012;8:13-8.

44. Cho H, Jiang Y, Li X, Deming M. The relationship between self-reported viral load suppression and quality of life among people living with HIV in South Carolina. AIDS Care. 2019;32:1-8.

45. Faria E, Gonçalves T, Carvalho F, Piccinini C. Longitudinal assessment of coping and quality of life over 24 months postpartum in mothers living with HIV. Journal of Health Psychology. 2019:135910531987743.

46. Khademi N, Saeidi S, Zangeneh A, Saeidi F, Choobtashani M. The Relationship between Life Satisfaction and Quality of Life among People Living with HIV/AIDS in Kermanshah‐West of Iran. Health & Social Care in the Community. 2020;28.

47. Reychler G, Caty G, Vincent A, Billo S, Jean cyr Y. Validation of the French version of the World Health Organization quality of life HIV instrument. PLoS One. 2013;8(9):e73180.

48. Veld DHI', Pengpid S, Colebunders R, Skaal L, K P. High-risk alcohol use and associated socio-demographic, health and psychosocial factors in patients with HIV infection in three primary health care clinics in South Africa. Int J STD AIDS. 2016;28(7):615-59.

49. Reis R, Haas V, Santos C, Teles S, Gir E. Symptoms of depression and quality of life of people living with HIV/AIDS. Rev Lat Am Enfermagem. 2011;19:874-81.
