# Supplementary figures and images for: Use of quality‐of‐life instruments for people living with HIV: a global systematic review and meta‐analysis
Source: J Int AIDS Soc. 2022 Apr 9;25(4):e25902. doi: 10.1002/jia2.25902 (PMC8994483; doi:10.1002/jia2.25902)

**Supplementary Figure 1. Funnel plot for included studies**

**
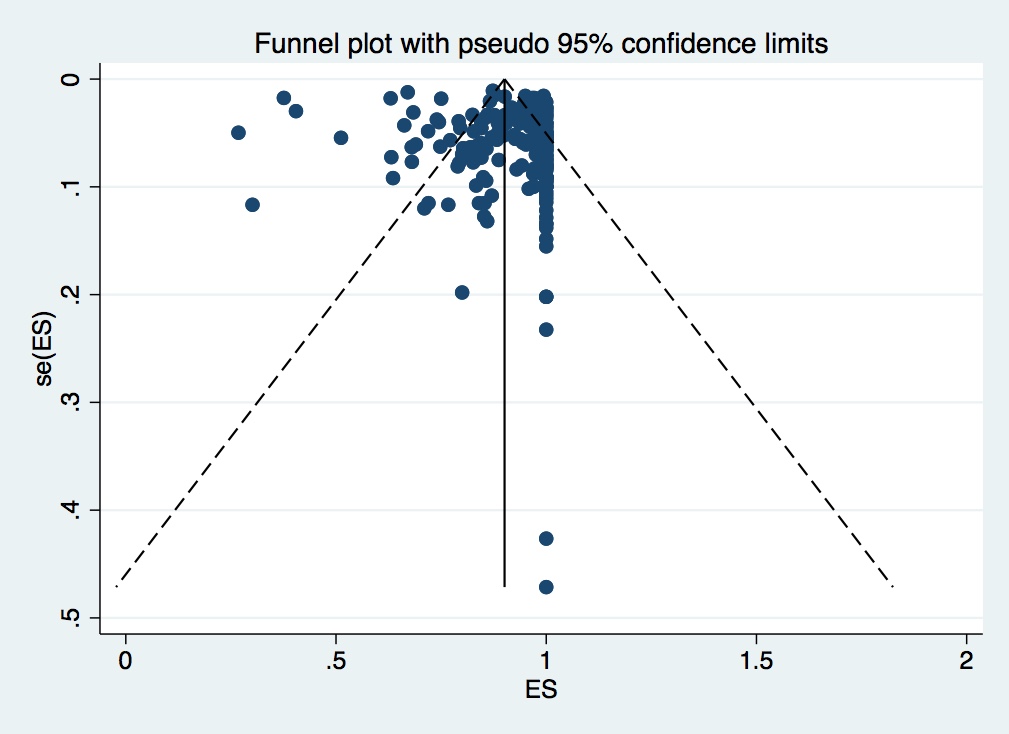
**

Egger’s test = 0.028

Supplement: Supplementary file 3 — Figure S1: Funnel plot for included studies. [file JIA2-25-e25902-s008.docx]

**Supplementary Figure 2. Funnel plot for included and imputed studies**


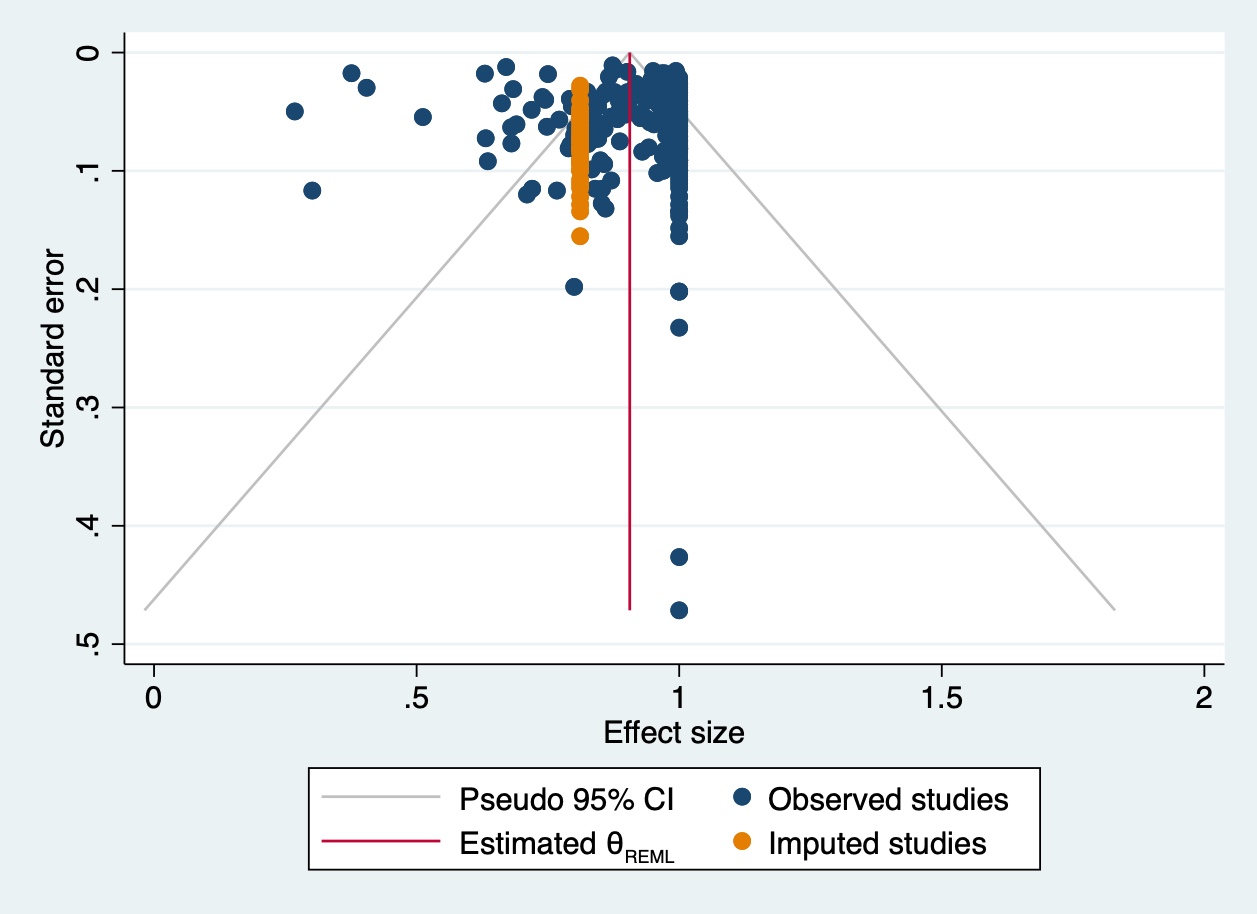

Supplement: Supplementary file 4 — Figure S2: Funnel plot for included and imputed studies. [file JIA2-25-e25902-s004.docx]
