# Supplementary material for: Use of quality‐of‐life instruments for people living with HIV: a global systematic review and meta‐analysis
Source: J Int AIDS Soc. 2022 Apr 9;25(4):e25902. doi: 10.1002/jia2.25902 (PMC8994483; doi:10.1002/jia2.25902)
Supplement: Supplementary file 5 — Table S1: Distribution of the most commonly used HRQoL instruments. [file JIA2-25-e25902-s005.docx]

**Supplementary Table 1. Distribution of the most commonly used HRQoL instruments**

|  | **WHOQOL- HIV BREF** | **MOS-HIV** | **SF- 36** | **EQ-5D** | **WHOQOL BREF** | **SF-12** | **HAT-QOL** | **Non- top 7** | **Total number** |
| --- | --- | --- | --- | --- | --- | --- | --- | --- | --- |
| **Country income level** |  |  |  |  |  |  |  |  |  |
| High | 28  (7.8%) | 61  (17.7%) | 58  (16.9%) | 41  (11.9%) | 11  (3.2%) | 43  (12.5%) | 15  (4.4%) | 87  (25.3%) | 344 |
| Upper-middle | 63  (25.7%) | 30  (13.0%) | 28  (12.1%) | 22  (9.5%) | 26  (11.3%) | 5  (2.2%) | 19  (8.2%) | 38  (16.5%) | 231 |
| Lower-middle | 37  (27.8%) | 16  (13.4%) | 6  (5.0%) | 12  (10.1%) | 19  (16.9%) | 6  (5.0%) | 7  (5.9%) | 16  (13.4%) | 119 |
| Low | 17  (22.4%) | 24  (38.1%) | 3  (4.8%) | 3  (4.8%) | 5  (7.9%) | 0  (0%) | 2  (3.2%) | 9  (14.3%) | 63 |
| Mix | 0 | 2  (15.4%) | 0 | 2  (15.4) | 0 | 1  (7.7) | 0 | 8  (61.5%) | 13 |
|  |  |  |  |  |  |  |  |  |  |
| **World Region** |  |  |  |  |  |  |  |  |  |
| African | 37  (22.0%) | 35  (21.3%) | 11  (6.7%) | 17  (10.4%) | 14  (8.5%) | 6  (3.7%) | 10  (6.1%) | 35  (21.3%) | 164 |
| Americas | 33  (13.3%) | 38  (15.3%) | 45  (18.1%) | 21  (8.5%) | 3  (1.2%) | 23  (9.3%) | 29  (11.7%) | 56  (22.6%) | 248 |
| Eastern Mediterranean | 5  (22.7%) | 2  (9.1%) | 6  (27.3%) | 1  (4.5%) | 4  (18.2%) | 0 | 0 | 4  (18.2%) | 22 |
| European | 21  (16.0%) | 24  (18.3%) | 20  (15.3%) | 22  (16.8%) | 6  (4.6%) | 12  (9.2%) | 1  (0.8%) | 25  (19.1%) | 131 |
| South-East Asian | 28  (38.4%) | 8  (11.0%) | 2  (2.7%) | 2  (2.7%) | 19  (26.0%) | 1  (1.4%) | 1  (1.4%) | 12  (16.4%) | 73 |
| Western Pacific | 22  (20.4%) | 22  (20.4%) | 9  (8.3%) | 13  (12.0%) | 15  (13.9%) | 9  (8.3%) | 1  (0.9%) | 17  (15.7%) | 108 |
| Mix | 0 | 4  (16.7%) | 2  (8.3%) | 4  (16.7%) | 0 | 4  (16.7%) | 1  (4.2%) | 9  (37.5%) | 24 |
|  |  |  |  |  |  |  |  |  |  |
| **Study Design** |  |  |  |  |  |  |  |  |  |
| RCT | 17  (15.5%) | 26  (23.6%) | 11  (10.0%) | 9  (8.2%) | 5  (4.5%) | 6  (5.5%) | 2  (1.8%) | 34 | 110 |
| Observational | 109  (21.6%) | 76  (15.0%) | 61  (12.1%) | 49  (9.7%) | 46  (9.1%) | 36  (7.1%) | 35  (6.9%) | 93 | 505 |
| Cohort | 19  (13.8%) | 31  (22.5%) | 21  (15.2%) | 11  (9.0%) | 10  (7.2%) | 12  (8.7%) | 6  (4.3%) | 28 | 138 |
| Economic Evaluation | 0 | 0 | 2  (11.8%) | 11  (64.7%) | 0 | 1  (5.9%) | 0 | 0 | 17 |
|  |  |  |  |  |  |  |  |  |  |
| **Study Setting** |  |  |  |  |  |  |  |  |  |
| Hospital | 88  (21.7%) | 67  (16.5%) | 48  (11.9%) | 43  (10.6%) | 35  (8.6%) | 31  (7.7%) | 19  (4.7%) | 74  (18.3%) | 405 |
| Community/ GP | 34  (15.5%) | 37  (16.9%) | 26  (11.9%) | 27  (12.3%) | 15  (6.8%) | 20  (9.1%) | 14  (6.4%) | 46  (21.0%) | 219 |
| NGO/ Peer-led | 5  (15.6%) | 7  (21.9%) | 4  (12.5%) | 1  (3.1%) | 4  (12.5%) | 1  (3.1%) | 3  (9,4%) | 7  (21.9%) | 32 |
| Others | 14  (16.7%) | 12  (14.3%) | 13  (15.5%) | 7  (8.3%) | 5  (6.0%) | 3  (3.6%) | 5  (6.0%) | 25  (29.8%) | 84 |
| Unclear | 4  (13.3%) | 10  (33.3%) | 4  (13.3%) | 2  (6.7%) | 2  (6.7%) | 0 | 2  (6.7%) | 6  (20.0% | 30 |
| **Target Population** |  |  |  |  |  |  |  |  |  |
| PLHIV vs non-PLHIV | 3  (3.8%) | 13  (16.7%) | 18  (23.1%) | 12  (15.4%) | 9  (11.5%) | 5  (6.4%) | 2  (2.6%) | 16  (20.5%) | 78 |
| PLHIV only | 142  (20.5%) | 127  (18.4%) | 77  (11.1%) | 68  (9.8%) | 52  (7.5%) | 50  (7.2%) | 41  (5.9%) | 135  (19.5%) | 692 |
| **Subpopulation Characteristics** |  |  |  |  |  |  |  |  |  |
| Women | 6  (8.8%) | 9  (13.2%) | 2  (2.9%) | 7  (10.3%) | 9  (13.2%) | 6  (8.8%) | 4  (5.9%) | 25  (36.8%) | 68 |
| MSM | 5  (17.9%) | 5  (17.9%) | 3  (10.7%) | 1  (3.4%) | 3  (10.7%) | 6  (21.4%) | 1  (3.6%) | 4  (14.3%) | 28 |
| Mixed population (except women and MSM) | 134  (19.9%) | 119  (17.7%) | 90  (13.3%) | 72  (10.7%) | 49  (7.3%) | 43  (6.4%) | 38  (5.6%) | 129  (19.1%) | 674 |
|  |  |  |  |  |  |  |  |  |  |
