# Supplementary material for: Use of quality‐of‐life instruments for people living with HIV: a global systematic review and meta‐analysis
Source: J Int AIDS Soc. 2022 Apr 9;25(4):e25902. doi: 10.1002/jia2.25902 (PMC8994483; doi:10.1002/jia2.25902)
Supplement: Supplementary file 6 — Table S2: Coding for the categorisation of the items. [file JIA2-25-e25902-s007.docx]

**Supplementary Table 2. Coding for the categorisation of the items**

| **Items category** | **All terms captured in that items category** |
| --- | --- |
| **General health** | general health, get sick a little easier than other people, I am healthy as anybody I know, I expect health to get worse, health is excellent, pleased with how healthy I have been, I have not been able to live the way I would like to because I am so worried about my health, I have been worried about my health getting worse, I have been worried about my CD4 count, somewhat ill, healthy as anybody. |
| **Mobility** | Mobility, bothered by any physical problems related to your HIV infection, limitations on moderate activities to health, limitations on climbing several stairs due to health, accomplished less due to physical health, limited work or other activities due to physical health, rate your general health, how much does your health limit your activities, typical activities (vigorous, moderate, walking uphill, lifting, walking, eating/using the toilet), satisfied with physical activity, physically limited in my ability to do routine household chores, self-care, extent of physical health problems. |
| **Usual activities** | Usual Activities (work, housework, family, leisure), Leisure activities, daily living activities, capacity for work, opportunity for leisure activities, Activities of daily living, work capacity, Participation in and opportunities for recreation/leisure activities, limited work or other activities due to physical/emotional problems, problems with work/activities as a result of emotional problems (such as feeling depressed/anxious), problems with work/activities due to physical activities, health keep you working at job/work around the house/going to school, unable to do certain kinds or amounts of work, housework, school because of your health, worried about not being able to do my job/routine daily activities as I have in the past, felt that having HIV has limited the amount of work I can do at my job/routine daily activities, does your health keep you working at job/work around the house/going to school. |
| **Pain** | Pain/discomfort, physical pain, bodily pain, pain interfere with normal work, pain has limited my ability to be physically active. |
| **Negative feelings** | Anxiety/depression, negative feelings such as blue mood, despair, anxiety, depression, fear of the future, Negative feelings, how you feel (nervous, feeling so down, downhearted/blue, worn out, tired), feelings (nervous, downhearted/blue, down in the dumps), Feel often (worn out, weighed down by your health problems, discouraged by health problems, despair over health problems, afraid because of your health), feeling bad, extent of emotional problems. |
| **Positive feelings** | Positive feelings, feeling calm/peaceful, how you feel (full of pep, calm/peaceful, energy, happy person), feelings (calm/peaceful, happy), Feel often (full of pep), enjoyed living, strong will to live, content with my life, control of my life, good about myself, motivated to do things. |
| **Energy & vitality** | Energy/Vitality, fatigue, sleep and rest, enough energy, tired, I have been too tired to be socially active. |
| **Cognitive/cognition** | Concentration, thinking/learning/memory and concentration, Time (difficult reasoning and solving problems, forget things that happened, trouble keeping your attention, difficulty doing activities involving concentration and thinking). |
| **Bodily appearance, self-esteem** | Bodily Appearance, Self-esteem, I like who I am. |
| **Medical treatment** | Medical Treatment need, Dependence on medicinal substances and medical aids, taking my medicine has made it hard to live a normal life, taking my medicine has made me feel better, taking my medicine has made me feel more sick than I think I am, taking my medicine has made me feel as if I am fighting HIV. |
| **Acceptance & support** | Acceptance/Support by others or self, social support, I have been able to accept the fact that I have HIV. |
| **HIV disclosure** | HIV Disclosure, I have limited what I tell others about myself, I have been afraid to tell other people that I have HIV, I have been worried about my family members finding out that I have HIV, I have been worried about people at my job/routine daily activities finding out that I have HIV, I have been worried that I will lose my source of income if other people find out that I have HIV. |
| **Relationships, social** | Personal Relationships, Social activities, social activities interfered by physical or emotional problems, satisfied with how socially active I am, my health has limited my social activities |
| **Sex life** | Sex Life, Sexual activity, satisfied with my sexual life, interested in sex, difficult to get sexually aroused. |
| **Safety/physical environment** | Safety/physical environment, transport, physical safety and security, home environment, Physical environment (pollution / noise / traffic / climate), Transport. |
| **Financial security** | Financial security, Financial resources: I have been worried about having to live on a fixed income, I have been worried about how to pay my bills, money has been too tight for me to care for myself the way I think I should, I have had enough money to do the things I enjoy doing. |
| **Access and quality of info/health services** | Access to info/health services, Health and social care: accessibility and quality, Opportunities for acquiring new information and skills, I have felt as if my doctor was someone who listens to me, I have felt confident in my doctor’s ability to care for people with HIV, I have felt certain that my doctor has my best interest in mind. |
| **Spirituality/religion** | Meaningful life, Spirituality, religion, personal beliefs. |
| **Blame for HIV status/stigma** | Stigma, Blame for HIV status. |
| **Death worry** | Death Worry, I have felt uncertain about what the future holds for me, I have been worried about when I am going to die, life planning. |
