# Supplementary material for: Use of quality‐of‐life instruments for people living with HIV: a global systematic review and meta‐analysis
Source: J Int AIDS Soc. 2022 Apr 9;25(4):e25902. doi: 10.1002/jia2.25902 (PMC8994483; doi:10.1002/jia2.25902)
Supplement: Supplementary file 7 — Table S3: Sub‐analysis of pooled completion rates of HRQoL instruments. [file JIA2-25-e25902-s002.docx]

**Supplementary Table 3. Sub-analysis of pooled completion rates of HRQoL instruments**

|  | Number of studies | % completed (95% CI) | *I^2^* (p value) |
| --- | --- | --- | --- |
| Overall | 233 | 95.9 (97.0-100) | 99.2 (<0.001) |
| **Country income level** |  |  |  |
| High | 100 | 92.2 (89.7-94.3) | 99.3 (p <0.001) |
| Upper-middle | 74 | 97.7 (95.3-993) | 99.2 (p <0.001) |
| Lower-middle | 33 | 99.4 (98.6-99.9) | 93.4 (p <0.001) |
| Low | 17 | 96.0 (92.9-98.3) | 97.7 (p <0.001) |
| Mix | 9 | 97.9 (94.6-99.7) | 99.3 (p <0.001) |
| **Region of the world** |  |  |  |
| South-East Asian | 19 | 99.2 (97.8-99.9) | 93.6 (p <0.001) |
| African | 39 | 98.3 (97.2-99.1) | 97.2 (p <0.001) |
| Americas | 76 | 94.8 (92.0-97.1) | 99.2 (p <0.001) |
| Eastern Mediterranean | 6 | 99.8 (93.7-100) | 91.0 (p <0.001) |
| Western Pacific | 47 | 95.4 (90.9-98.4) | 99.5 (p <0.001) |
| Europe | 39 | 91.9 (88.5-94.8) | 99.3 (p <0.001) |
| Middle East and North African | 1 | 100 (85.8-100) | - |
| Mix | 6 | 97.1 (92.0-99.7) | 98.8 (p <0.001) |
| **Study design** |  |  |  |
| Cross-sectional | 153 | 97.1 (95.9-98.2) | 99.2 (<0.001) |
| Randomised controlled trial | 28 | 97.1 (94.3-99.0) | 97.3 (<0.001) |
| Case-control | 3 | 100 (100-100) | - |
| Cohort | 48 | 88.7 (94.7-97.0) | 99.3 (<0.001) |
| **Name of instrument** |  |  |  |
| WHOQOL-BREF | 24 | 98.1 (89.8-100) | 99.7 (<0.001) |
| MOS-HIV | 53 | 97.0 (95.6-98.2) | 97.1 (<0.001) |
| Mixed | 15 | 93.1 (88.8-96.5) | 98.5 (<0.001) |
| SF-6D | 1 | 71.9 (67.4-76.1) | - |
| WHOQOL-HIV | 45 | 97.5 (95.6-98.9) | 98.0 (<0.001) |
| SF-36 | 34 | 94.7 (89.3-98.4) | 99.1 (<0.001) |
| HAT-QOL | 9 | 98.7 (95.0-100) | 95.1 (<0.001) |
| SF-12 | 27 | 89.9 (84.3-94.3) | 99.5 (<0.001) |
| EQ-5D | 22 | 94.7 (91.9-97.0) | 99.1 (<0.001) |
| SF-21 | 1 | 98.5 (97.5-99.2) | - |
| WHOQOL-STI-BREF | 1 | 100 (95.8-100) | - |
| **Instrument type** |  |  |  |
| Generic | 112 | 94.6 (92.2-96.5) | 99.5 (<0.001) |
| HIV-specific | 104 | 97.3 (96.2-98.2) | 97.5 (<0.001) |
| Mix | 17 | 95.1 (91.0-98.0) | 98.7 (<0.001) |
| **Number of items*** |  |  |  |
| 4-21 | 58 | 93.4 (90.6-95.7) | 99.4 (<0.001) |
| 24-31 | 66 | 97.4 (94.5-99.4) | 99.4 (<0.001) |
| 32-35 | 48 | 97.0 (95.4-98.2) | 96.9 (<0.001) |
| 36-120 | 56 | 95.4 (92.4-97.7) | 98.9 (<0.001) |

* separated by quartile
