# Supplementary material for: Use of quality‐of‐life instruments for people living with HIV: a global systematic review and meta‐analysis
Source: J Int AIDS Soc. 2022 Apr 9;25(4):e25902. doi: 10.1002/jia2.25902 (PMC8994483; doi:10.1002/jia2.25902)
Supplement: Supplementary file 8 — Table S4: Meta‐regression results of completion rate of HRQoL instruments. [file JIA2-25-e25902-s006.docx]

**Supplementary Table 4. Meta-regression results of completion rate of HRQoL instruments**

| Variable | Univariable | | | | Multivariable | | |  |
| --- | --- | --- | --- | --- | --- | --- | --- | --- |
|  | β (95% CI) | P-value | Adjusted R^2^ | β (95% CI) | | P-value | Adjusted R^2^ | |
| Country Income level |  |  | 10.5% |  | |  | 16.0% | |
| High | Reference |  |  | Reference | |  |  | |
| Upper-Middle | 0.066 (0.028 to 0.104) | 0.001 |  | 0.038 (-0.012 to 0.089) | | 0.135 |  | |
| Lower-Middle | 0.101 (0.053 to 0.149) | <0.001 |  | 0.087 (0.008 to 0.166) | | 0.031 |  | |
| Low | 0.061 (0.001 to 0.121) | 0.046 |  | 0.016 (-0.085 to 0.116) | | 0.754 |  | |
| Mix | 0.086 (0.010 to 0.161) | 0.027 |  | 0.037 (-0.067 to 0.141) | | 0.481 |  | |
| Region of the world |  |  | 4.5% |  | |  |  | |
| African | Reference |  |  | Reference | |  |  | |
| Americas | -0.061 (-0.109 to -0.013) | 0.013 |  | -0.019 (-0.096 to 0.058) | | 0.628 |  | |
| Mediterranean | -0.001 (-0.138 to 0.138) | 0.995 |  | 0.002 (-0.149 to 0.154) | | 0.975 |  | |
| Europe | -0.076 (-0130 to -0.023) | 0.005 |  | -0.047 (-0.131 to 0.038) | | 0.279 |  | |
| Middle East | 0.035 (-0.418 to 0.489) | 0.879 |  | 0.0 (-0.473 to 0.473) | | 1.000 |  | |
| Mix | -0.006 (-0.105 to 0.094) | 0.913 |  | -0.037 (-0.164 to 0.091) | | 0.572 |  | |
| South-East Asian | 0.014 (-0.056 to 0.084) | 0.700 |  | -0.014 (-0.097 to 0.069) | | 0.740 |  | |
| Western Pacific | -0.051 (-0.104 to 0.002) | 0.057 |  | -0.024 (-0.099 to 0.050) | | 0.521 |  | |
| Study design |  |  | 7.2% |  | |  |  | |
| Cohort | Reference |  |  | Reference | |  |  | |
| Cross-sectional | 0.085 (0.046 to 0.125) | <0.001 |  | 0.088 (0.045 to 0.132) | | <0.001 |  | |
| Randomised controlled trial | 0.083 (0.023 to 0.142) | 0.007 |  | 0.085 (0.020 to 0.151) | | 0.011 |  | |
| Case-control | 0.145 (-0.067 to 0.357) | 0.180 |  | 0.146 (-0.084 to 0.376) | | 0.213 |  | |
| Study setting |  |  | 0.4% |  | |  |  | |
| Hospital | Reference |  |  |  | |  |  | |
| General practice | 0.025 (-0.014 to 0.065) | 0.208 |  | 0.017 (-0.023 to 0.058) | | 0.394 |  | |
| Non-government organization | 0.050 (-0.020 to 0.120) | 0.158 |  | 0.0 (-0.083 to 0.084) | | 0.994 |  | |
| Other | 0.016 (-0.042 to 0.075) | 0.585 |  | 0.003 (-0.060 to 0.066) | | 0.921 |  | |
| Name of instrument |  |  | 4.5% |  | |  |  | |
| WHOQOL-BREF | Reference |  |  | Reference | |  |  | |
| MOS-HIV | 0.033 (-0.029 to 0.096) | 0.296 |  | 0.036 (-0.104 to 0.177) | | 0.610 |  | |
| Mixed | -0.008 (-0.088 to 0.073) | 0.854 |  | -0.063 (-0.246 to 0.121) | | 0.501 |  | |
| SF-6D | -0.198 (-0.438 to 0.043) | 0.106 |  | -0.230 (-0.492 to 0.031) | | 0.084 |  | |
| WHOQOL-HIV | 0.030 (-0.035 to 0.094) | 0.363 |  | 0.080 (-0.051 to 0.211) | | 0.229 |  | |
| SF-36 | -0.025 (-0.093 to 0.044) | 0.476 |  | -0.037 (-0.171 to 0.097) | | 0.587 |  | |
| HAT-QOL | 0.051 (-0.052 to 0.154) | 0.333 |  | 0.029 (-0.129 to 0.187) | | 0.718 |  | |
| SF-12 | -0.052 (-0.122 to 0.018) | 0.146 |  | -0.069 (-0.202 to 0.064) | | 0.307 |  | |
| EQ-5D | 0.009 (-0.063 to 0.080) | 0.813 |  | -0.029 (-0.171 to 0.114) | | 0.692 |  | |
| SF-21 | 0.068 (-0.161 to 0.298) | 0.559 |  | 0.072 (-0.190 to 0.333) | | 0.589 |  | |
| WHOQOL-STI-BREF | 0.083 (-0.223 to 0.389) | 0.592 |  | 0.125 (-0.206 to 0.455) | | 0.458 |  | |
| Instrument type |  |  | 4.0% |  | |  |  | |
| Generic | Reference |  |  | Reference | |  |  | |
| HIV-specific | -0.050 (-0.083 to -0.015) | 0.005 |  | 0.025 (-0.092 to 0.142) | | 0.678 |  | |
| Mix | -0.026 (-0.089 to 0.038) | 0.425 |  | 0.104 (-0.040 to 0.249) | | 0.156 |  | |
| Number of items |  |  | 0.8% |  | |  |  | |
| 4-21 | Reference |  |  | Reference | |  |  | |
| 24-31 | 0.028 (-0.016 to 0.073) | 0.211 |  | -0.096 (-0.219 to 0.027) | | 0.127 |  | |
| 32-35 | 0.045 (-0.004 to 0.094) | 0.069 |  | -0.022 (-0.115 to 0.071) | | 0.641 |  | |
| 36-120 | 0.006 (-0.041 to 0.053) | 0.808 |  | -0.021 (-0.135 to 0.094) | | 0.719 |  | |
